# Canagliflozin Markedly Decreased Proteinuria in a Patient With IgA Nephropathy

**DOI:** 10.7759/cureus.19446

**Published:** 2021-11-10

**Authors:** Muhammad Aameish, Milica Jovanovic, Mala A John, Donald Baumstein

**Affiliations:** 1 Internal Medicine/Nephrology, New York Medical College, Metropolitan Hospital Center, New York City, USA; 2 Nephrology, New York Medical College, Metropolitan Hospital Center, New York City, USA

**Keywords:** covid-19, sodium-glucose cotransporter-2 (sglt2) inhibitors, proteinuria, iga nephropathy, canagliflozin

## Abstract

IgA nephropathy (IgAN) remains one of the most common forms of glomerulonephritis, especially in developed countries with a low prevalence of infectious diseases. Despite supportive measures that slow the rate of progression of chronic kidney disease (CKD) in IgAN, many patients still progress to end-stage kidney disease. Proteinuria has been shown to be an adverse prognostic factor in IgAN. Data support the use of proteinuria reduction as a reasonably likely surrogate endpoint for a treatment’s effect on progression to end-stage renal disease (ESRD) in IgAN. Currently employed immunosuppressive strategies lack conclusive efficacy data, while there is evidence for treatment-induced toxicity. The current standard of care for the management of IgAN is intensive goal-directed supportive care. Recently the role of sodium-glucose cotransporter 2 (SGLT2) inhibitors in decreasing proteinuria and progression of CKD is widely being recognized. In this case report, we present a 44-year-old male with proteinuria and biopsy-proven IgAN who remained in remission after six months of steroids using the Pozzi protocol. He developed proteinuria five years after remission. At this point, canagliflozin was added to his angiotensin-receptor blocker (ARB) therapy resulting in a significant reduction in his proteinuria. Our case report may intrigue researchers to look into the role of canagliflozin in decreasing albuminuria in non-diabetic kidney disease, thus slowing the progression to ESRD.

## Introduction

Sodium-glucose cotransporter 2 (SGLT2) inhibitors have been used to substantially reduce CKD progression and decrease albuminuria. Dapagliflozin has been shown to be effective in both diabetic and non-diabetic CKD. The Dapagliflozin and Prevention of Adverse Outcomes in Chronic Kidney Disease Trial (DAPA-CKD) included 4,304 participants with glomerular filtration rate 25 to 75 mL per minute per 1.73 m2 and urine albumin:creatinine (alb:cr) ratio 200-5,000 mg/g. This study demonstrated that in patients with CKD with or without diabetes mellitus, the risk of decline of at least 50% in estimated glomerular filtration rate (eGFR), ESRD, death from renal or cardiovascular causes was significantly lower with dapagliflozin than with placebo [1]. A pre-specified analysis of the DAPA-CKD trial, which encompassed 270 patients with a diagnosis of IgA nephropathy (IgAN), now provides early evidence that dapagliflozin may be a safe and effective addition to the current standard of care in IgAN [2].

Canagliflozin also safely reduces kidney and cardiovascular events in people with type 2 diabetes and severely increased albuminuria [3]. In the Canagliﬂozin and Renal Events in Diabetes with Established Nephropathy Clinical Evaluation (CREDENCE) study, canagliﬂozin reduced the risk of kidney failure and cardiovascular outcomes in patients with type 2 diabetes and CKD (eGFR 30 to <90 mL/min/1.73 m^2^ and urinary alb:cr ratio >300-5,000 mg/g) who were already receiving an angiotensin-converting enzyme inhibitor (ACEi) or ARB [4]. In these studies, involving dapagliflozin and canagliflozin, patients were all on ACEi or ARB at baseline of the study. Here, we present a case where adding canagliflozin to preexisting ARB therapy markedly reduced albuminuria in non-diabetic kidney disease, specifically IgAN. In our literature review, we did not find canagliflozin studied in patients with IgAN.

## Case presentation

A 44-year-old Hispanic man presented in 2014 with a history of hypertension and had a urine protein (pr): creatinine (cr) ratio of 1.34 with 5-10 red blood cells per high power field on urine microscopy. His BUN was 14 and serum creatinine 0.7 mg/dL. Serological workup was negative. Kidney biopsy revealed IgAN with mesangial proliferative and sclerosing changes with mild to moderate activity and mild chronicity. By the updated 2017 Oxford classification of IgAN, he had M1, E0, S1, T0, C2 where M is Mesangial hypercellularity, E is endocapillary proliferation, S is segmental sclerosis, T is tubular atrophy and interstitial fibrosis and C is crescents. In addition, he had mild arteriolosclerosis.

**Figure 1:**
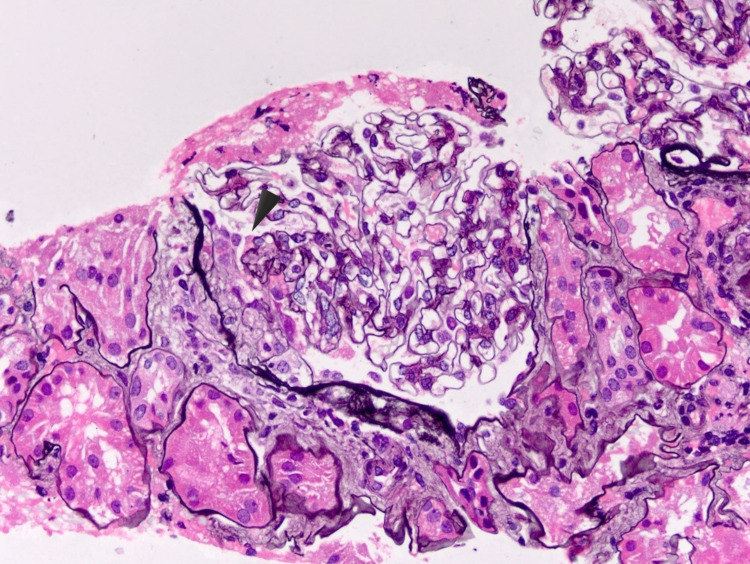
Patient's kidney biopsy from 2014 showing fibrocellular crescent.

After a four-month trial of an ARB, losartan, titrated to the highest dose, he still had urine pr:cr ratio of 1.16. He then received the Pozzi protocol consisting of six months of corticosteroids with three-day boluses of intravenous solumedrol on months 1, 3, and 5 and prednisone 0.5 mg per kg body weight on alternate days throughout the six month period. He went into remission with a decrease of urine pr:cr ratio to 0.19 within four months by March 2015.

He remained in remission on losartan 100 mg while tested with semiannual urine pr:cr ratios through October 2019. In March 2020, he acquired a COVID-19 infection but was not hospitalized. At an outside laboratory, his urine alb:cr ratio increased to 555 mg/g in August 2020. As noted in Table 1, in November 2020, his urine alb:cr ratio was 428 mg/g with a urine pr:cr ratio of 0.8. His then ARB (valsartan) dose was increased. One month later, we prescribed dapagliflozin 10 mg as a treatment for IgAN given his rising proteinuria, but he took canagliflozin due to cost considerations. Three months after starting the canagliflozin, his urine pr:cr ratio decreased to 0.3, a 62% reduction in proteinuria.

**Table 1:** Investigations of the patient

Labs/treatment	10/2019	03/2020	08/2020	11/2020	12/2020	3/2021
BUN- mg/dl	13	Covid Infection		18		16
Cr -mg/dl	0.91			0.8		0.9
GFR-ml/min/1.73m^2^	104			106		104
Urinary alb (mg): Cr (g)	60		555	428		132
Urine Pr:Cr ratio	0.16			0.8		0.3
HTN – mmHg	126/78			142/96		127/78
Treatment	Losartan 100 mg			Valsartan 80 mg increased to 160 mg	Canagliflozin started	

## Discussion

IgAN is one of the most common forms of glomerulonephritis, which despite the use of renin-angiotensin-aldosterone-system blockers and immunosuppressants, often progresses to kidney failure [2]. There is a large unmet need for treatment options for IgAN. The pre-specified analysis of the DAPA-CKD study demonstrates that in patients with IgAN, when added to ACEi or ARB therapy, dapagliflozin significantly and substantially reduces the risk of CKD progression with a favorable safety profile [2]. In this prespecified analysis, the primary outcome (decline in estimated GFR of at least 50%, ESKD, death from renal or cardiovascular causes) occurred in 4% in the dapagliflozin group compared to 15% in the placebo group over a median follow-up of 2.1 years. The dapagliflozin group had a hazard ratio of 0.29 and an absolute risk decline of 10.7% compared with the placebo. Median baseline urinary alb:cr ratio was 900 mg/g. Albuminuria declined 35% by month 4 and was sustained throughout follow-up.

In the CREDENCE trial, the median baseline urine alb:cr ratio was 927 mg/g. In addition, the relative kidney benefits were consistent over the entire range of albuminuria levels studied. Canagliflozin reduced efficacy outcomes for all urine albumin to creatinine ratio levels with no evidence that relative benefits varied between levels [3]. The subgroup with alb:cr ratio of less than 1000 mg/g in this secondary analysis had a median baseline albuminuria of 489 mg/g creatinine with an absolute reduction of 163 mg/g or a relative reduction of 35%. Our patient had a normal eGFR with albuminuria of 428 mg/g creatinine and 0.8 g of proteinuria/g creatinine. This was a similar amount of albuminuria as the subgroup with less than 1,000 mg albumin/g creatinine with type 2 diabetes in CREDENCE. However, his albuminuria decreased from 428 mg/g to 132 mg/g a relative reduction of 69%.

Although this decrease in albuminuria and proteinuria with canagliflozin in IgAN is a new finding, the conclusions drawn from this case report can only be considered as interesting. This is an evaluation based on a single patient and we have only one data point after starting canagliflozin. In addition, the ARB dose was also increased prior to measuring the effect of canagliflozin on urine protein and urine albumin. We believe the ARB would have played a minor role here since the ARB offered a minimal decrease in proteinuria after four months of use at the time of his initial IgAN diagnosis. Proteinuria is a surrogate endpoint and not the clinically more meaningful important endpoint of reducing the risk of kidney failure used in the DAPA-CKD and CREDENCE studies. However, in IgAN, there is a strong relationship between the level and duration of proteinuria and loss of kidney function [5]. There is also an association between proteinuria reduction and CKD progression in IgAN. In fact, many members of the Kidney Health Initiative workgroup concluded that data support the use of proteinuria reduction as a reasonably likely surrogate endpoint for a treatment’s effect on progression to ESKD in IgAN [5]. The ongoing EMPA-KIDNEY trial (ClinicalTrials.gov Identifier: NCT03594110) has recruited a large population of CKD patients and is likely to provide further information on the effectiveness and safety of SGLT2 inhibitors in patients with IgAN.

It is possible that COVID-19 may have played a role in the relapse of IgAN in this patient leading to proteinuria. Huang et al. report increased proteinuria and gross hematuria as a consequence of exacerbation of a patient's biopsy-proven IgAN concurrent with a COVID-19 infection [6]. Abramson et al. report newly diagnosed IgAN in a kidney biopsy after a man presented with gross hematuria and proteinuria 24 hours after a COVID-19 vaccine [7]. In addition, there are two recent reports [8,9] of three patients with prior biopsy-proven IgAN presenting with gross hematuria usually with increased proteinuria within 24 hours of the second dose of SARS-CoV-2 vaccine. Once the vaccine is injected, mRNA encoding prefusion-stabilized spike glycoprotein is translated by host cells, and major histocompatibility complex molecules subsequently present viral protein segments to elicit immune responses with antibody production, including IgG/A/M. Importantly, the receptor-binding domain of the viral spike protein is the immunodominant target of neutralizing antibodies in both infected patients [10] and vaccinated persons [11]. This supports the idea that both COVID-19 infection and vaccine may play a role in the activation of IgAN. It is interesting to consider that Canagliflozin may also be effective in reducing proteinuria post-COVID-19 Infection secondary to underlying glomerulonephritis.

## Conclusions

Dapagliflozin has demonstrated decreased albuminuria and renal protection in patients with IgA nephropathy. Canagliflozin has demonstrated similar results in patients with CKD and type 2 diabetes with severely increased albuminuria. In our case presented, a patient with known IgA nephropathy and a recent increase in albuminuria and proteinuria had a 69% decline in albuminuria after the addition of canagliflozin to his ARB. This highlights the potential of canagliflozin acting to offer renal protection in IgA nephropathy if borne out by the further study. The association of COVID-19 and this patient’s increased albuminuria in the context of current case reports call attention to the consideration of the possible role of canagliflozin ameliorating COVID-19-induced glomerulonephritis.
